# Recommendations for Prehospital Airway Management in Patients with Suspected COVID-19 Infection

**DOI:** 10.5811/westjem.2020.5.47540

**Published:** 2020-06-15

**Authors:** James Hart, Rebecca Tracy, Matthew Johnston, Sara Brown, Connor Stephenson, Jason Kegg, James Waymack

**Affiliations:** * Southern Illinois University School of Medicine, Department of Emergency Medicine, Springfield, Illinois; † Memorial Medical Center, Springfield, Illinois; ‡ Southern Illinois University School of Medicine, Springfield, Illinois; § State of Illinois EMS Medical Director, Department of Public Health, Springfield, Illinois

## Abstract

In light of the rapid spread of coronavirus disease 2019 (COVID-19) across the United States, the Centers for Disease Control and Prevention (CDC) and hospitals nationwide have developed new protocols to address infection control as well as the care of critical patients. Airway management has been particularly difficult; the challenge of quickly establishing an airway in patients must be balanced by the risk of aerosolizing respiratory secretions and putting the provider at risk of infection. Significant attention has been given to developing protocols for the emergency department and critical care units, but little guidance regarding establishing airway and respiratory support for patients in the prehospital setting has been made available. While some of the recommendations can be extrapolated from hospital guidelines, other factors such as environment and available resources make these protocols unfeasible. Through review of current literature the authors established recommendations regarding airway management and the provision of respiratory support to patients developing respiratory failure related to COVID-19.

## INTRODUCTION

Since the discovery of the novel human coronavirus (now SARS-CoV-2, commonly referred to as COVID-19) was reported to the World Health Organization on December 31, 2019, our understanding of the pathophysiology and treatment of the disease has rapidly evolved.^1^ As of February 11, 14% of COVID-19 infections in China have been classified as severe and 5% as critical, with patients developing respiratory failure.^2^ As early as March 4, Chinese health officials estimate the case fatality rate of COVID-19 at 3.7%.^3^ Current recommendations for airway management in patients with respiratory failure have targeted in-hospital providers (emergency physicians, critical care physicians, and anesthesiologists) and have neglected to provide guidance for prehospital providers who may find themselves making these critical decisions without on-scene oversight.^3^–^7^ It is our goal to provide recommendations for emergency medical service (EMS) providers regarding establishing airway protection/respiratory support for patients with suspected COVID-19 infection.

## PERSONS OF INTEREST AND INITIAL ASSESSMENT

As EMS is the likely first point of contact that many potential COVID-19 patients have with the healthcare system, it is important for EMS providers to be able to identify patients in whom COVID-19 infection should be suspected. Screening of patients for possible person under investigation (PUI) status should begin with 911 dispatch and other public safety answering points. Screening for COVID-19 infection should include the following: history of foreign travel or travel to current hotspots identified by the Centers for Disease Control and Prevention; close contact (less than six feet for more than 10 minutes) of a known COVID-19 positive patient or PUI; or to a person with a flu-like illness with worsening dyspnea, body aches, sore throat, non-productive cough, and/or gastrointestinal symptoms.^8^ If no screening information has been provided, precautions should be taken when responding to any patient who reports dyspnea or flu-like illness. A surgical mask should be placed on the patient to minimize possible spread of infection as the first step in airway management to protect EMS providers. Suspicion of a possible PUI by dispatch, or after on-scene evaluation, should be communicated to the receiving hospital to allow for adequate preparations for the patient’s arrival to the emergency department, especially if the need for a definitive airway appears imminent.

## SUMMARY OF CURRENT IN-HOSPITAL RECOMMENDATIONS

Current recommendations for in-hospital providers regarding patients who develop respiratory failure involve endotracheal intubation using the rapid sequence intubation (RSI) technique and video laryngoscopy (VL) with minimal use of bag-valve mask (BVM) ventilation.^3^–^5^ BVM use should be limited to minimize aerosol generation as a potential source of viral exposure.^4^,^5^ For patients with normal airway, awake intubation should be avoided and modified RSI is strongly recommended. Sufficient paralysis should be assured before intubation to decrease the likelihood of aerosolizing infectious respiratory secretions as well as to prevent aspiration/vomiting.^9^ As previously shown, apneic oxygenation can prevent desaturation and should be implemented; however, it is important to take into consideration that the use of non-rebreather (NRB) or nasal cannula increases the risk of aerosolizing viral particles.^4^,^10^,^11^ A surgical mask should be placed over the NRB or nasal cannula to limit contamination of the environment. VL has also been shown in previous studies to be superior to direct laryngoscopy (DL) for first-pass success rate.^12^ Therefore, to minimize exposure and maximize first-pass success, it is currently recommended to use VL for intubation. Following intubation, a HEPA filter is attached directly to the endotracheal tube and then attached to the ventilator.

The use of noninvasive positive pressure ventilation (NIV) in patients with suspected COVID-19 infections is controversial. While some observations have shown that high-flow nasal cannula has shown improvements in oxygenation and decreased rates of intubation, there have been no reported changes in mortality.^4^ NIV is associated with increased aerosolization of respiratory secretions and, if there is an improper seal, the risk of contaminating the work environment is significantly increased. Of the bedside therapies used to support oxygenation and ventilation, clinical experience taken from the 2012 SARS coronavirus outbreak suggests that intubation, BVM manual ventilation, bedside suction, and non-invasive ventilation pose the highest airborne droplet exposure risks.^13^ It was also observed by Brewster et al that patients on NIV have a high rate of failure (76%), requiring intubation.^4^ This also mirrors findings from a multicenter cohort of 302 patients with Middle East respiratory syndrome coronavirus, in which 92% of patients treated with bilevel positive airway pressure failed this modality and required intubation.^14^ Therefore, consideration must be taken that most patients will fail NIV and if resources allow, patients should be considered for endotracheal intubation as the initial intervention, which will also provide source control as long as the ventilator circuit is intact.

Supraglottic airway (SGA) devices pose a unique challenge in dealing with the spread of COVID-19. While they are superior to NIV, they do not provide the same quality of seal compared to that of endotracheal intubation.^4^,^5^ Overinflation with bag ventilation could potentially aerosolize respiratory secretions, increasing the risk of spreading infection to healthcare providers as well as contaminating the workspace. However, second-generation SGAs (LMA ProSeal, intubating LMA Fastrach, laryngeal tube, laryngeal tube LTS II, Combitube, and Easytube) have been shown to have improved seal compared to their first-generation counterparts.^4^,^15^–^17^ In light of the current situation, EMS providers in Seattle, Washington, have already begun using I-gel SGAs with HEPA filters in the field for respiratory arrest or failure in the event of failed RSI.^18^,^19^

## PREHOSPITAL RECOMMENDATIONS

First and foremost, appropriate personal protective equipment (PPE) should be worn at all times while providing care for patients with a suspected COVID-19 infection. This should include a N95 mask or powered air purifying respirator (if not available, use a surgical mask), gloves, gown, and eye protection (minimum of glasses with temple shield). A surgical mask should be placed on the patient as soon as possible to prevent further contamination of the workspace with infectious respiratory droplets.^8^,^20^

If first responders have access to VL and are able to perform RSI, then endotracheal intubation in the field should be attempted. Preoxygenation with nasal cannula and non rebreather mask should be performed, making sure to cover with a surgical mask as well as leaving the nasal cannula in place while attempting intubation. Providers should only make a single attempt at endotracheal intubation, as multiple unsuccessful attempts will result in repeated, unnecessary exposure to potentially infectious respiratory droplets. Avoid DL in these cases for similar reasons. If a patient has impending respiratory failure and providers do not have access to VL or RSI, they should proceed immediately to SGA insertion with a second-generation device.

Following successful ET or SGA placement, a HEPA filter should be immediately attached *directly* to the ET tube or SGA (see Figure 1 for details). This will prevent contamination of upstream equipment, such as capnography, tubing, and the bag-valve apparatus. Failure to do so could also result in contamination of the monitor, as capnography is not rated to filter out viral particles.^21^

Basic Life Support (BLS) crews may find themselves in a challenging predicament, as in some districts and states BLS crews are unable to use SGAs. The current American Heart Association guidelines allow for the use of BVM for BLS resuscitation with a tight seal and a HEPA filter while wearing appropriate PPE.^22^ BVM should only be used if unable to implement ET tube or SGA. In this scenario a two-person technique is recommended to provide the best seal as well as implementation of appropriate airway adjuncts (nasal pharyngeal airway, oral pharyngeal airway, etc). Caution should be taken to prevent over-inflation of the lungs. Plans to transfer care to an Advanced Life Support (ALS) crew or intercept should be implemented immediately.

## CONCLUSION

The rapid spread of COVID-19 has posed unique challenges to prehospital providers with regard to airway management. Using appropriate PPE is essential in avoiding unnecessary exposure to prehospital providers. The placement of a surgical mask, as soon as logistically possible, on the patient and HEPA filter use with any airway adjuncts is crucial to prevent potential spread of infectious respiratory droplets. Important changes to the usual algorithm for intubating patients involves avoiding DL in favor of VL, the use of RSI if available, and progression to second-generation SGA if endotracheal intubation attempt fails or if RSI/VL is not available. By following these recommendations, prehospital providers will be able to minimize their risk of contracting COVID-19 infection while providing high-quality care for their critical patients.
